# The Cardiocerebral Resuscitation protocol for treatment of out-of-hospital primary cardiac arrest

**DOI:** 10.1186/1757-7241-20-65

**Published:** 2012-09-15

**Authors:** Gordon A Ewy

**Affiliations:** 1 University of Arizona Sarver Heart Center, University of Arizona, Tucson, AZ, 85704, USA

**Keywords:** Ventricular fibrillation, Resuscitation, Cardiac arrest, Cardiopulmonary resuscitation, Cardiocerebral resuscitation, Primary cardiac arrest, Emergency medical system, Out-of-hospital cardiac arrest

## Abstract

Out-of-hospital cardiac arrest (OHCA) is a significant public health problem in most westernized industrialized nations. In spite of national and international guidelines for cardiopulmonary resuscitation and emergency cardiac care, the overall survival of patients with OHCA was essentially unchanged for 30 years--from 1978 to 2008 at 7.6%. Perhaps a better indicator of Emergency Medical System (EMS) effectiveness in treating patients with OHCA is to focus on the subgroup that has a reasonable chance of survival, e.g., patients found to be in ventricular fibrillation (VF). But even in this subgroup, the average survival rate was 17.7% in the United States, unchanged between 1980 and 2003, and 21% in Europe, unchanged between 1980 and 2004. Prior to 2003, the survival of patients with OHCA, in VF in Tucson, Arizona was less than 9% in spite of incorporating previous guideline recommendations. An alternative (non-guidelines) approach to the therapy of patients with OHCA and a shockable rhythm, called Cardiocerebral Resuscitation, based on our extensive physiologic laboratory studies, was introduced in Tucson in 2003, in rural Wisconsin in 2004, and in selected EMS areas in the metropolitan Phoenix area in 2005. Survival of patients with OHCA due to VF treated with Cardiocerebral Resuscitation in rural Wisconsin increased to 38% and in 60 EMS systems in Arizona to 39%. In 2004, we began a statewide program to advocate chest compression-only CPR for bystanders of witnessed primary OHCA. Over the next five years, we found that survival of patients with a shockable rhythm was 17.7% in those treated with standard bystander CPR (mouth-to-mouth ventilations plus chest compression) compared to 33.7% for those who received bystander chest-compression-only CPR. This article on Cardiocerebral Resuscitation, by invitation following a presentation at the 2011 Danish Society Emergency Medical Conference, summarizes the results of therapy of patients with primary OHCA treated with Cardiocerebral Resuscitation, with requested emphasis on the EMS protocol.

## Background

Out-of-hospital cardiac arrest (OHCA) is a significant public health problem in most westernized industrialized nations [1]. In spite of national and international guidelines for cardiopulmonary resuscitation (CPR) and emergency cardiac care, the overall survival of patients with OHCA was essentially unchanged for 30 years—from 1978 to 2008 at 7.6% [2]. A better indicator of an emergency medical system’s (EMS) effectiveness in treating patients with OHCA is to focus on the subgroup that has a reasonable chance of survival; patients found to be in ventricular fibrillation (VF) [3]. But even in this subgroup, the average reported survival rate was 17.7% in the United States, unchanged between 1980 and 2003, and 21% in Europe, unchanged between 1980 and 2004 [4,5]. Following the 2005 guidelines, the Resuscitation Outcomes Consortium, arguably representing some of the better EMS in the United States and Canada, reported that the median survival rates for patients with OHCA due to VF arrest managed with the 2005 guidelines had a survival rate of 22% [6]. However there was wide variability in survival observed by the reporting sites, ranging from 7.7% to 39.9% [6]. This uneven survival shows that following the same approach (in this case the 2005 national and international Guidelines for CPR and Emergency Cardiac Care) does not assure similar survival of patients with OHCA.

Because many believe that resuscitation for cardiac arrest guidelines should be predominantly based on randomized control trials in man, guideline changes were rarely influenced by basic experimental studies. In fact, in 2000, the weight of evidence for animal studies was markedly decrease by the American Heart Association Guidelines for CPR and ECC [7,8].

In our experimental resuscitation laboratory, where the animals were anesthetized but not paralyzed, we found that survival was improved following several minutes of untreated ventricular fibrillation (VF) arrest by emphasizing chest compression only CPR (CO-CPR) and a new approach for advanced cardiac support [9].

The poor and stagnant survival rates of patients with OHCA due to VF arrest in our community led us to conclude that survival would be improved by applying in man the techniques that improved survival of VF arrest in our physiologic research laboratory [9,10].

**Figure 1:**
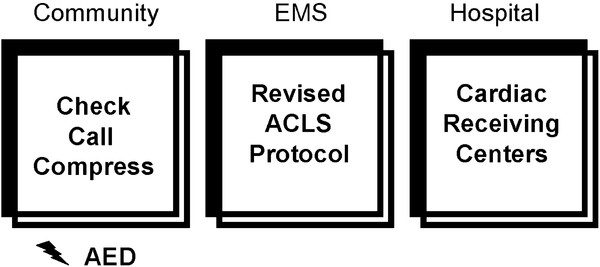
**The three components of Cardiocerebral Resuscitation for primary cardiac arrest; the Community component consists of “Check” to see if the person has a cardiac arrest, “Call” to activate the Emergency Medical Services (EMS), and “Compress” for chest compression-only CPR.** If an automated external defibrillator (AED) is readily available, its use should be encouraged. The **EMS** component consists of a revised advanced cardiac life support protocol (ACLS). The **Hospital** component is a hospital that has been designated as a Cardiac Receiving Center or equivalent. *Figure reproduced from the Journal of the American College of Cardiology (JACC), with permission.*

**Figure 2:**
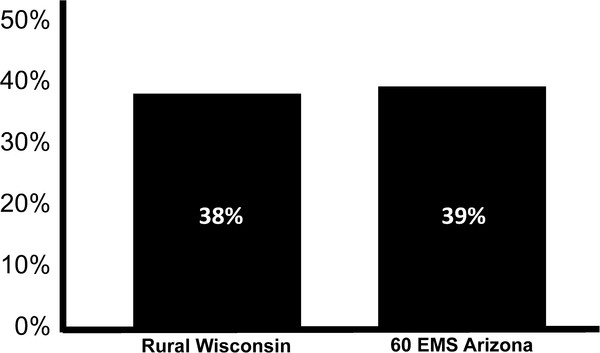
**Survival of patients with out-of-hospital cardiac arrest due to ventricular fibrillation treated with EMS component of Cardiocerebral Resuscitation in rural Wisconsin and 60 Emergency Medical Systems (EMS) in Arizona **[13,15].

The three components of Cardiocerebral Resuscitation are the **Community**, the **EMS** (emergency medical system), and the **Hospital** (Figure 1). In 2003 we instituted the Community and EMS components of Cardiocerebral Resuscitation for patients with primary OHCA and a shockable rhythm [9-11]. These components of Cardiocerebral Resuscitation were subsequently introduced in rural Wisconsin in 2004 and in some areas of Arizona in 2005 [12-15]. Each was willing to try a new approach, as their survival rates of patients with OHCA were poor and not improved in spite of changes made with each national guideline’s update. Each subsequently reported improved survival (38% in rural Wisconsin and 39% in 60 EMS in Arizona) (Figure 2) [13-15].

Cardiocerebral Resuscitation is advocated only for primary cardiac arrest. Note that it is not only for adults but also for younger individuals (such as those due to Commotio Cordis or congenital heart abnormalities) that result in primary cardiac arrest; an unexpected witnesses collapse in an individual who is not responsive. It is not recommended for cardiac arrest secondary to respiratory failure from drowning, drug overdose, or advanced pulmonary disease. Here chest compressions and assisted ventilations are necessary [16].

This new approach to primary cardiac arrest eliminated the term “pulmonary”, because of its initial lack of emphasis on mouth-to-mouth ventilation and prompt intubation and assisted positive pressure ventilation [9]. Cardiocerebral Resuscitation emphasis is on near constant blood flow to the heart and the brain by near continuous chest compressions throughout resuscitation attempts [9]. Assisted ventilation is initially not necessary at the onset of primary cardiac arrest, as the patient was breathing normally at the moment of the arrest. Therefore the arterial blood is oxygenated. Since the blood is essentially not circulating prior to initiation of chest compressions, the arterial blood remains oxygenated for several minutes [17].

The requirement for mouth-to-mouth ventilation as the first step of bystander resuscitation prevented most from initiation bystander CPR.

## The community component of cardiocerebral resuscitation

The first component of Cardiocerebral Resuscitation is the Community (Figure 1) as the early initiation of bystander chest compressions significantly improves the chances of survival [18]. The public is encouraged to “Check, Call, Compress.” If an automated external defibrillator (AED) is readily available, they are encouraged to use it as well (Figure 1) [19].

Essential to the “Check” is to determine if the patient has a primary cardiac arrest. Our Sarver Heart Center Resuscitation Research Group thinks this can best be accomplished by teaching that a primary cardiac arrest is an unexpected witnessed (seen or heard) collapse in an individual who is not responsive. An important part of the “Check” is to recognize gasping. Gasping, except in newborns is a sign of cardiac arrest and is common during the first few minutes of primary cardiac arrest [20,21]. Gasping during resuscitation efforts is a sign of adequate blood flow to the brain stem. Survival is greater in patients who are gasping [22].

**Figure 3:**
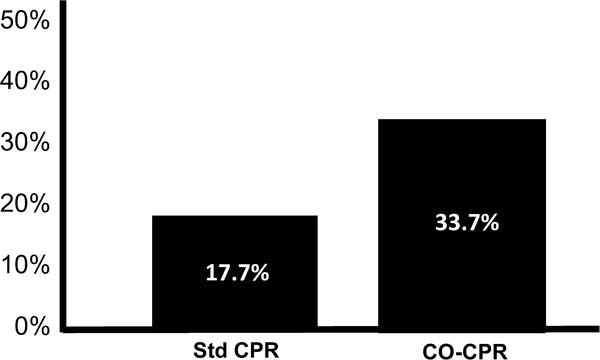
**Survival to hospital discharge in Arizona of patients with out-of-hospital cardiac arrest between the beginning of 2005 and the end of 2009 who received bystander guidelines recommended standard cardiopulmonary (Std-CPR) or compression only cardiopulmonary (CO-CPR) **[23].

With the cooperation of the Arizona Bureau of Emergency Medical Services Department, of the Arizona Medical Services and Trauma Systems, of the Arizona Department of Health Services, and the SHARE (Save Hearts in Arizona Registry and Education program) share@arizona.gov under the direction of Bentley J. Bobrow, M.D., a statewide effort was instituted in Arizona in 2004 to teach and advocate chest compression only CPR (CO-CPR) for bystanders of witnessed primary cardiac arrest. The result was doubling of survival of patients with out-of-hospital cardiac arrest (Figure 3) [23].

## The EMS component of cardiocerebral resuscitation

The EMS or emergency medical service component of Cardiocerebral Resuscitation advocates a revised sequence of interventions for patients with primary cardiac arrest, not witnessed by EMS providers. This component advocates the prompt initiation of 200 continuous chest compressions prior to and immediately after a single indicated direct current shock, altered airway management to avoid prolonged interruptions of chest compressions and hyperventilation, and the early administration of epinephrine (adrenalin).

### Defibrillation first for an observed, unexpectedly primary cardiac arrest

The individual with a witnessed unexpected collapse who is not responsive is usually in the electrical phase of ventricular fibrillation arrest. The most important intervention is prompt defibrillation. The highest survival rates of individuals with out-of-hospital cardiac arrest was reported from the casinos in Las Vegas where our colleagues taught the security guards to recognize primary cardiac arrest and to use an automated external defibrillator (AED) [24]. Those individuals with a witnessed arrest who were shocked within 3 minutes had a survival rate of 74% and those shocked after 3 minutes had a survival rate of 49% [24]. Thus if the patient’s arrest is witnessed, the initial step for EMS personnel is prompt defibrillation.

### Chest compressions prior to defibrillation

For individuals no longer in the Weisfeldt and Becker’s “electrical phase” (the first four or so minutes) of VF arrest, chest compressions prior to defibrillation is recommended to decompress and to perfuse the heart [25]. With cardiac arrest, the arterial pressure falls and the venous pressure rises, until Guyton’s mean circulatory filling pressure, is reached. Professor Stig Steen reported, in open chest swine, with the onset of ventricular fibrillation, as blood shifts from the high pressure arterial system into the lower pressure venous system, the right ventricle volume increases while the left ventricular volume decreases [26]. We showed the same phenomenon with MRI imaging [27].

**Figure 4:**
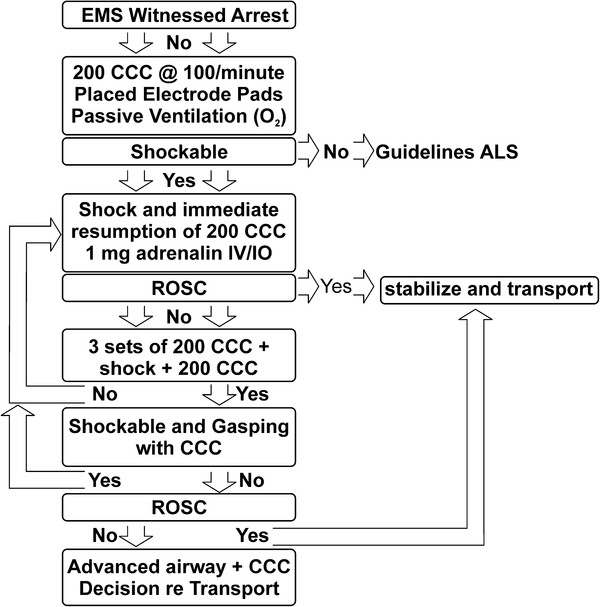
**The Emergency Medical Services (EMS) protocol of Cardiocerebral Resuscitation.** CCC is continuous chest compressions, O2 is oxygen, ACLS is advanced cardiac life-support, IV/IO is intravenous or interosseous, ROSC is return of spontaneous circulation.

In cardiac arrest, the prompt reestablishment of cardiac and cerebral perfusion is of paramount importance. The sternum should be compressed at least 2 inches at a rate of 100 times per minute [28,29]. Each chest compression must be followed by full chest wall recoil [30]. Therefore, if good bystander chest compressions are not underway, the first EMS intervention is the prompt initiation of 200 uninterrupted chest compressions at an optimal rate, depth, and recoil (Figure 4) [9,29,31].

Whenever possible, two EMS individuals are assigned as “chest compressors” to take up their position on the opposite sides of the patient’s chest. The individuals providing chest compressions are encouraged to alternate every minute and, in the absence of a chest compression rate and depth detector, the other person is assigned the task of continuously monitoring the rate and the quality of chest compressions. Ideally, metronomes provide audible prompts of 100 per minute. Studies have indicated that while bystanders tend to compress too slowly, emergency medical service (EMS) personnel tend to compress too rapidly [32,33]. The optimal compression rate in man is probably near 100/min, but in experimental animals we found that the cardiac output increases with rates up to 120/min [33]. A recent editorial reviewing the currently available literature, concluded that, “the sweet spot for manual chest compressions is a rate of about 120 per minute” [34]. None of these studies were with continuous chest compression CPR.

### Airway management

Airway management initially consists of passive ventilation by the insertion of an oral pharyngeal or supraglottic airway, and the provision of high flow oxygen via a non-rebreather mask (Figure 4) [13]. This approach eliminates the two most common deleterious effects of endotracheal intubation (ETI) during cardiac arrest; prolonged interruptions of chest compressions and hyperventilation [35].

We delayed ETI by EMS, based on our observations during resuscitation efforts of patients with in-hospital cardiac arrest. We often observed prolonged interruptions of chest compressions while physicians accomplished ETI. We assumed that EMS providers had similar difficulties. Subsequently, Wang and associates documented that paramedic ETI for OHCA interrupts chest compressions for prolonged periods [36]. They reported on 100 patients with OHCA; the median duration of first ETI was 47 seconds, almost one-third exceeded one minute and one-fourth exceeded 3 minutes [36]. Interruptions of chest compressions for these durations during cardiac arrest are usually lethal. Eliminating the need for early ETI also frees up that EMS person to quickly apply the defibrillator pads, to turn on the defibrillator, and to perform other essential duties such as relieving the person doing chest compressions or providing the early administration of epinephrine (Figure 4) [29]. Intubation is usually performed following ROSC to stabilize the patient’s airway during transportation.

### Preventing deleterious effects of hyperventilation

Passive ventilation also prevents “Death by Hyperventilation.” [35] Following cardiac arrest, rapid, forceful positive pressure ventilations increase intrathoracic pressures, thereby decreasing venous return to the chest and heart [37]. Unfortunately increases in intrathoracic pressures are transmitted, probably via increasing the spinal fluid pressures, to an increase in intracranial pressures, which decreases blood flow to the brain [38].

### Each shock is immediately followed by 200 chest compressions

Following each defibrillation shock, EMS personnel are prohibited from looking at the electrocardiogram (EKG) or palpating for an arterial pulse (Figure 4) [29]. The reason for this is that following prolonged untreated VF, a direct current shock will usually terminate the VF but will result in pulseless electrical activity (PEA) [39]. In fact this is the way we produce PEA in the experimental physiologic laboratory. Ventricular fibrillation is induced but is not treated for 8 to 12 minutes. Electrical defibrillation then results in classic PEA, and a normal or near normal electrocardiographic waveform, but a blood pressure so low that it cannot be palpated [39]. Medical personnel, not familiar with this phenomenon, observe the normal sinus rhythm on the EKG monitor and assume that the defibrillation shock has not only restored the sinus rhythm, but also the blood pressure. So viewing the resultant sinus rhythm on the EKG display, they do not resume chest compressions, but begin searching for a pulse. However PEA untreated usually deteriorates into heart block [39]. Rather than resume chest compressions, the previous response was often to try to get a pacemaker—without resumption of chest compressions, the patient’s rhythm further deteriorates into asystole. Then resuscitation efforts would be resumed, but not for VF arrest, but for asystolic arrest where survival is unusual. In contrast the immediate resumption of chest compressions following an effective defibrillation shock improves coronary perfusion pressure and most often leads to an effective perfusion pressure.

In patients with VF arrest, after three cycles of “200 CCC, rhythm analysis, plus shock are completed,” if the patients is not gasping, ventilation is no doubt indicated (Figure 4) [29].

### The role of epinephrine

Based on findings in our and others experimental laboratories, early epinephrine administration is recommended [9,40,41]. To administer epinephrine early, intraosseous injection is often necessary [40]. The late administration of epinephrine in man late in the cardiac arrest has not been shown to improve survival [42,43]. Although the incidence of ROSC is improved, survival is not.

The only randomized, controlled, double-blind trial of epinephrine vs. placebo in man, analyzed by a Bayesian interpretation of the results, suggest a benefit of epinephrine (CI 2.1 with 95% credible interval of 0.8 to 6.6) [44,45].

## The hospital component

The third component of Cardiocerebral Resuscitation (Figure 1) is the “Hospital” component. In Arizona, hospitals are designated as “Cardiac Receiving Centers” once they have committed to providing optimal care to resuscitated but comatose cardiac arrest patients. In Arizona, it is acceptable for the EMS ambulance to bypass hospitals to transport patients with OHCA to a designated Cardiac Receiving Center. Multivariant analysis did not find an association between transport time and survival [46,47]. Cardiac Receiving Center hospitals have the trained staff, the necessary equipment, the expertise and the commitment to provide optimal care, including therapeutic mild hypothermia and urgent cardiac catheterization “24/7” for patients who attain ROSC (most of whom are comatose) following cardiac arrest [48,49].

## Future perspectives

There are areas of the world that have excellent survival rates following the national and international Guidelines, for example Seattle, Washington and Northern Netherlands [50,51]. Both of these areas have a high incidence of bystander CPR and a record of dispatch assisted CPR (DA-CPR). A meta-analysis of randomized control trials of DA-CPR has shown that survival of patients with OHCA is improved with dispatch assisted chest-compression only CPR [52]. These facts and the improved survival in Arizona where CO-CPR was and is advocated, supports the contention that one of the more important interventions is the early initiation of bystander CPR.

## Conclusions

Out-of-hospital cardiac arrest is a major public health problem. In spite of recurrent updates of guidelines for CPR and ECC, survival of patients treated according to the guidelines is quite uneven, relatively good in a few areas, but poor in most. In contrast, everywhere that Cardiocerebral Resuscitation has been instituted survival of the subset of patients with the greatest chance of survival, those with an OHCA and a shockable rhythm has dramatically improved.

Every EMS system needs know the survival rate of those with OHCA and the best chance of survival—a primary cardiac arrest and a shockable rhythm. Knowing the survival rate of all patients with OHCA is not helpful as most such patients have little or no chance of survival, and leads to a defeatist mentality by EMS.

Therefore, in the authors view, if the survival rate of such patients with primary OHCA and a shockable rhythm in your community is less than 30%, you should seriously consider instituting Cardiocerebral Resuscitation for patients with primary cardiac arrest,

It is difficult to improve what you don’t measure.

## Competing interest

Dr. Ewy is one of the principal investigators of a "HeartRescue" grant from the Medtronic's Foundation to the University of Arizona. The purpose of this grant is to improve survival of patients with cardiac arrest.

## References

[B1] Lloyd-Jones DM Hong Y Labarthe D Defining and setting national goals for cardiovascular health promotion and disease reduction: the American Heart Association's strategic Impact Goal through 2020 and beyond Circulation 2010 121 4 586 613 10.1161/CIRCULATIONAHA.109.192703 20089546

[B2] Sasson C Rogers MA Dahl J Kellermann AL Predictors of survival from out-of-hospital cardiac arrest: a systematic review and meta-analysis Circ Cardiovasc Qual Outcomes 2010 3 63 81 10.1161/CIRCOUTCOMES.109.889576 20123673

[B3] Chamberlain D Cummins RO Abramson N Recommended guidelines for uniform reporting of data from out-of-hospital cardiac arrest: The Utstein style Resuscitation 1991 22 1 1 26 10.1016/0300-9572(91)90061-3 1658890

[B4] Rea TD Eisenberg MS Sinibaldi G White RD Incidence of EMS-treated out-of-hospital cardiac arrest in the United States Resuscitation 2004 63 17 24 10.1016/j.resuscitation.2004.03.025 15451582

[B5] Atwood C Eisenberg MS Herlitz J Rea TD Incidence of EMS-treated out-of-hospital cardiac arrest in Europe Resuscitation 2005 67 75 80 10.1016/j.resuscitation.2005.03.021 16199289

[B6] Nichol G Thomas E Callaway CW Regional variation in out-of-hospital cardiac arrest incidence and outcome JAMA 2008 300 1423 1431 10.1001/jama.300.12.1423 18812533 PMC3187919

[B7] American Heart Association Guidelines 2000 for cardiopulmonary resuscitation and emergency cardiovascular care: international consensus on science Circulation 2000 102 (I) I-1 I-34810964901

[B8] Ewy GA Cardiocerebral resuscitation should replace cardiopulmonary resuscitation for out-of-hospital cardiac arrest Curr Opin Crit Care 2006 12 189 192 10.1097/01.ccx.0000224859.25217.5b 16672774

[B9] Ewy GA Cardiocerebral resuscitation: the new cardiopulmonary resuscitation Circulation 2005 111 2134 2142 10.1161/01.CIR.0000162503.57657.FA 15851620

[B10] Kern KB Valenzuela TD Clark LL An alternative approach to advancing resuscitation science Resuscitation 2005 64 261 268 10.1016/j.resuscitation.2004.08.009 15733752

[B11] Ewy GA A new approach for out-of-hospital CPR: a bold step forward Resuscitation 2003 58 271 272 10.1016/S0300-9572(03)00268-5 12969602

[B12] Kellum MJ Kennedy KW Ewy GA Cardiocerebral resuscitation improves survival of patients with out-of-hospital cardiac arrest Am J Med 2006 119 335 340 10.1016/j.amjmed.2005.11.014 16564776

[B13] Kellum MJ Kennedy KW Barney R Cardiocerebral Resuscitation Improves Neurologically Intact Survival of Patients With Out-of-Hospital Cardiac Arrest Ann Emerg Med 2008 52 244 252 10.1016/j.annemergmed.2008.02.006 18374452

[B14] Garza AG Gratton MC Salomone JA Lindholm D McElron J Archer R Improved patient survival using a modified resuscitation protocol for out-of-hospital cardiac arrest Circulation 2009 119 2597 2605 10.1161/CIRCULATIONAHA.108.815621 19414637

[B15] Bobrow BJ Ewy GA Clark L Passive oxygen insufflation is superior to bag-valve-mask ventilation for witnessed ventricular fibrillation out-of-hospital cardiac arrest Ann Emerg Med 2009 54 656 662 10.1016/j.annemergmed.2009.06.011 19660833

[B16] Berg RA Wilcoxson D Hilwig RW The need for ventilatory support during bystander CPR Ann Emerg Med 1995 26 342 350 10.1016/S0196-0644(95)70084-6 7661426

[B17] Berg RA Kern KB Hilwig RW Assisted ventilation does not improve outcome in a porcine model of single-rescuer bystander cardiopulmonary resuscitation Circulation 1997 95 6 1635 1641 10.1161/01.CIR.95.6.1635 9118534

[B18] Rea TD Helbock M Perry S Increasing use of cardiopulmonary resuscitation during out-of-hospital ventricular fibrillation arrest: survival implications of guideline changes Circulation 2006 114 2760 2765 10.1161/CIRCULATIONAHA.106.654715 17159062

[B19] Weisfeldt ML Kerber RE McGoldrick RP Public access to defibrillation. The Automatic Defibrillation Task Force Am J Emerg Med 1996 14 7 684 692 10.1016/S0735-6757(96)90090-X 8906771

[B20] Zuercher M Ewy GA Gasping during cardiac arrest Curr Opin Crit Care 2009 15 185 188 10.1097/MCC.0b013e3283298e00 19276800

[B21] Zuercher M Ewy GA Otto CW Gasping in response to basic resuscitation efforts: observation in a Swine model of cardiac arrest Critical Care Research and Practice 2010 1 1 7 10.1155/2010/351638 PMC2951081 20948884

[B22] Bobrow BJ Zuercher M Ewy GA Gasping during cardiac arrest in humans is frequent and associated with improved survival Circulation 2008 118 2550 2554 10.1161/CIRCULATIONAHA.108.799940 19029463 PMC2707277

[B23] Bobrow BJ Spaite DW Berg RA Chest compression-only CPR by lay rescuers and survival from out-of-hospital cardiac arrest JAMA 2010 304 1447 1454 10.1001/jama.2010.1392 20924010

[B24] Valenzuela T Roe D Nichol G Clark L Spaite D Hardman R Outcomes of rapid defibrillation by security officers after cardiac arrest in casinos New Eng J Med 2000 343 1206 1209 10.1056/NEJM200010263431701 11071670

[B25] Weisfeldt ML Becker LB Resuscitation after cardiac arrest: a 3-phase time-sensitive model JAMA 2002 288 3035 3038 10.1001/jama.288.23.3035 12479769

[B26] Steen S Liao Q Pierre L Paskevicius A Sjoberg T The critical importance of minimal delay between chest compressions and subsequent defibrillation: a haemodynamic explanation Resuscitation 2003 58 249 258 10.1016/S0300-9572(03)00265-X 12969599

[B27] Sorrell VL Bhatt RD Berg RA Cardiac magnetic resonance imaging investigation of sustained ventricular fibrillation in a swine model–with a focus on the electrical phase Resuscitation 2007 73 2 279 286 10.1016/j.resuscitation.2006.08.032 17241733

[B28] Ewy GA Alternative approaches to external chest compression Circulation 1986 74 6 Pt 2 IV98 101 3536168

[B29] Ewy GA Kellum MJ Paradis NA, Halperin HR, Karn KB, Venzel V, Chamberlain DA Cardiocerebral resuscitation: a new approach to out-of-hospital cardiac arrest Cardiac Arrest: The Science and Practice of Resuscitation Medicine, 2nd Edition Cambridge University Press 2007 747 756

[B30] Aufderheide TP Pirrallo RG Yannopoulos D Incomplete chest wall decompression: a clinical evaluation of CPR performance by EMS personnel and assessment of alternative manual chest compression-decompression techniques Resuscitation 2005 64 353 362 10.1016/j.resuscitation.2004.10.007 15733766

[B31] Ewy GA Kern KB Recent advances in cardiopulmonary resuscitation: cardiocerebral resuscitation J Am Coll Cardiol 2009 53 149 157 10.1016/j.jacc.2008.05.066 19130982

[B32] Abella BS Sandbo N Vassilatos P Chest compression rates during cardiopulmonary resuscitation are suboptimal: a prospective study during in-hospital cardiac arrest Circulation 2005 111 428 434 10.1161/01.CIR.0000153811.84257.59 15687130

[B33] Feneley MP Maier GW Kern KB Influence of compression rate on initial success of resuscitation and 24 hour survival after prolonged manual cardiopulmonary resuscitation in dogs Circulation 1988 77 240 250 10.1161/01.CIR.77.1.240 3335070

[B34] Nolan JP Perkins GD Soar J Chest Compression Rate: Where is the Sweet Spot? Circulation 2012 125 2968 2970 10.1161/CIRCULATIONAHA.112.112722 22623716

[B35] Aufderheide TP Lurie KG Death by hyperventilation: a common and life-threatening problem during cardiopulmonary resuscitation Crit Care Med 2004 32 S345 351 10.1097/01.CCM.0000134335.46859.09 15508657

[B36] Wang HE Simeone SJ Weaver MD Callaway CW Interruptions in cardiopulmonary resuscitation from paramedic endotracheal intubation Ann Emerg Med 2009 54 645 652 10.1016/j.annemergmed.2009.05.024 19573949

[B37] Aufderheide TP The problem with and benefit of ventilations: should our approach be the same in cardiac and respiratory arrest? Curr Opin Crit Care 2006 12 207 212 10.1097/01.ccx.0000224863.55711.56 16672778

[B38] Yannopoulos D Nadkarni VM McKnite SH Intrathoracic pressure regulator during continuous-chest-compression advanced cardiac resuscitation improves vital organ perfusion pressures in a porcine model of cardiac arrest Circulation 2005 112 6 803 811 10.1161/CIRCULATIONAHA.105.541508 16061732

[B39] Ewy GA Defining electromechanical dissociation Ann Emerg Med 1984 13 830 832 10.1016/S0196-0644(84)80452-7 6476549

[B40] Zuercher M Kern KB Indik JH Epinephrine improves 24-hour survival in a swine model of prolonged ventricular fibrillation demonstrating that early intraosseous is superior to delayed intravenous administration Anesth Analg 2011 112 884 890 10.1213/ANE.0b013e31820dc9ec 21385987

[B41] Otto CW Yakaitis RW Ewy GA Effect of epinephrine on defibrillation in ischemic ventricular fibrillation Am J Emerg Med 1985 3 285 291 4004996 10.1016/0735-6757(85)90048-8

[B42] Olasveengen TM Sunde K Brunborg C Thowsen J Steen PA Wik L Intravenous drug administration during out-of-hospital cardiac arrest: a randomized trial JAMA 2009 302 2222 2229 10.1001/jama.2009.1729 19934423

[B43] Haqihara A Hasegawa M Abe T Nagata T Wakata Y Miyazaki S Prehospital epinephrine use and survival among patients with out-of-hospital cardiac arrest JAMA 2012 307 1161 1168 10.1001/jama.2012.294 22436956

[B44] Jacobs IG Finn JC Jelinek GA Oxer HF Thompson PL Effect of adrenaline on survival in out-of-hospital cardiac arrest: A randomised double-blind placebo-controlled trial Resuscitation 2012 83 e105 21745533 10.1016/j.resuscitation.2011.06.029

[B45] Youngquist ST Niemann JT Regarding"Effect of adrenaline on survival in out-of-hospital cardiac arrest: A randomised double-blind placebo-controlled trial Resuscitation 2012 83 e105 22266068 10.1016/j.resuscitation.2011.09.035

[B46] Spaite DW Bobrow BJ Vadeboncoeur TF The impact of prehospital transport interval on survival in out-of-hospital cardiac arrest: implications for regionalization of post-resuscitation care Resuscitation 2008 79 1 61 66 10.1016/j.resuscitation.2008.05.006 18617315

[B47] Spaite DW Stiell IG Bobrow BJ Effect of transport interval on out-of-hospital cardiac arrest survival in the OPALS study: implications for triaging patients to specialized cardiac arrest centers Ann Emerg Med 2009 54 248 255 10.1016/j.annemergmed.2008.11.020 19167783

[B48] Bobrow BJ Kern KB Regionalization of postcardiac arrest care Curr Opin Crit Care 2009 15 221 227 10.1097/MCC.0b013e328329c293 19469023

[B49] Bobrow BJ Vadeboncoeur TF Clark L Chikani V Establishing Arizona's statewide cardiac arrest reporting and educational network Prehosp Emerg Care 2008 12 381 387 10.1080/10903120802100670 18584508

[B50] Becker L Gold LS Eisenberg M White L Hearne T Rea T Ventricular fibrillation in King County, Washington: A 30-year perspective Resuscitation 2008 79 22 27 10.1016/j.resuscitation.2008.06.019 18687513

[B51] Berdowski J Blom MT Bardai A Tan HL Tijssen JGP Koster RW Impact of onsite or dispatched automated external defibrillator use on survival after out-of-hospital cardiac arrest Circulation 2011 124 2225 2232 10.1161/CIRCULATIONAHA.110.015545 22007075

[B52] Hupfl M Selig HF Nagele P Chest-compression-only versus standard cardiopulmonary resuscitation: a meta-analysis Lancet 2010 376 1552 1557 10.1016/S0140-6736(10)61454-7 20951422 PMC2987687

